# A Role Theory Perspective on How and When Goal-Focused Leadership Influences Employee Voice Behavior

**DOI:** 10.3389/fpsyg.2018.01244

**Published:** 2018-07-25

**Authors:** Jing Qian, Xiaoyan Li, Bin Wang, Baihe Song, Wei Zhang, Meng Chen, Yi Qu

**Affiliations:** ^1^Department of Human Resource Management, Business School, Beijing Normal University, Beijing, China; ^2^School of Business, Jiangxi Normal University, Nanchang, China

**Keywords:** goal-focused leadership, voice, reward omission, punishment omission, coworker helping and support, role theory

## Abstract

Despite an increasing number of studies that identify leaders’ role in promoting employees’ voice behavior, little is known about the role that supervisors’ goal-focused leadership plays in this. The current study aims to address this research gap by using the role theory to explain how supervisors’ goal-focused leadership influences employees’ voice behavior and the conditions under which supervisors’ have maximum impact on employee voice. A field study of 197 employees and their immediate supervisors offered support for our model. The results indicated a positive association between goal-focused leadership and employees’ voice behavior that was mediated by leaders’ omission of reward and punishments. We also found that perceived helping and support from coworkers positively moderated the relationship between leaders’ reward and punishment omission and employees’ voice behavior such that the relationship was weaker when coworker helping and support was higher. The findings provide more comprehensive picture of the process by which goal-focused leadership influences employee voice and highlight how coworkers can buffer the negative effect of ineffective managerial reward and punishment omission. The practical implications of this research, its limitations and directions for future research are also discussed.

## Introduction

Voice behavior is defined as “informal and discretionary communication by an employee of ideas, suggestions, concerns, information about problems, or opinions about work-related issues to persons who might be able to take appropriate action, with the intent to bring about improvement or change” (Morrison, 2014, p. 172). Unlike task performance or in-role behavior, voice is generally understood as a type of “above and beyond” behavior that is essential to the adaptation and success of organizations (Morrison, 2014). Increasing recognition of the importance of voice behavior has led scholars to recognize voice as being associated with both individuals and organizations. When employees engage in this upward communication behavior, they are challenging the status quo and may initiate changes to established work arrangements and processes, which may enhance their perception of control, increase overall job satisfaction and motivation and decrease stress (Greenberger and Strasser, 1986; Parker and Asher, 1993; Morrison and Milliken, 2000). Organizations also benefit from voice behavior because it contributes to effective organizational decision making, organizational learning, innovation and better error detection (Argyris and Schon, 1978; Locke et al., 1981; Nemeth and Kwan, 1985; Enz and Schwenk, 1991; Morrison and Milliken, 2000).

Although there are benefits that flow from voice behavior, recent research indicates that engaging in voice behavior can be draining, and employees are often very hesitant to use their voice (Milliken et al., 2003; Detert and Treviño, 2010). Voice behavior is commonly regarded as risky and associated with anxiety and stress, which may account for the tendency toward silence (Morrison, 2011, 2014). Given that voice behavior does not happen automatically and is not cost-free, organizations and leaders should actively promote and encourage voice behavior at work. Morrison (2011) concludes that when employees have a clear perception of their role, in other words, they understand clearly what is expected of them in their job, they are more likely to engage in voice behavior. This is because employees who have clear role expectations can avoid the negative outcomes of role ambiguity, such as anxiety and confusion, generated by role ambiguity (Kahn et al., 1964; Rizzo et al., 1970). Not surprisingly, supervisors are considered to play an important part in helping followers clarify role expectations. Previous studies have identified a range of contextual antecedents of voice behavior, such as transformational leadership, ethical leadership, leaders’ openness, leader–member exchange and group voice climate and caring climate (Detert and Burris, 2007; Botero and Van Dyne, 2009; Liang et al., 2012; Tangirala and Ramanujam, 2012; Wang and Hsieh, 2013).

One contextual factor thought to play a prominent role in organizations’ success is goal-focused leadership, which is defined as a leadership style characterized by an emphasis on goal achievement that is supported by the setting appropriate goals, suggesting means and providing task structure and feedback (Colbert and Witt, 2009). Not surprisingly then, goal focused leadership has been found to serve as a situational cue for better employee performance (Colbert and Witt, 2009; Kim et al., 2017). In line with the emerging literature indicating a relationship between goal-focused leadership and follower outcomes (e.g., Colbert and Witt, 2009; Perry et al., 2010; Kim et al., 2017), we investigated the relationship between goal-focused leadership and voice behavior amongst employees working in a logistics company located in northern China.

Although prior studies have led to great advances in understanding of the effects of goal-focused leadership on follower outcomes (Colbert and Witt, 2009; Perry et al., 2010; Kim et al., 2017), the approaches applied to date have limitations. Previous studies have used trait activation theory (Tett and Guterman, 2000), conservation of resources theory (Hobfoll, 1989), role making processes theory (Graen, 1976), and resource allocation theory (Kanfer and Ackerman, 1989) to explain the consequences of goal-focused leadership (Colbert and Witt, 2009; Perry et al., 2010; Kim et al., 2017). These studies all assume that goal-focused leadership can help employees to improve and thus deliver desirable outcomes. For example, Colbert and Witt (2009) used trait activation theory to argue that goal-focused leadership helps subordinates behave more conscientiously and thus perform more effectively. Previous scholars seem to have ignored the possibility that goal-focused leadership may help leaders to improve and in turn enhance follower performance. Addressing this research gap, we have drawn on role theory (Rizzo et al., 1970; Biddle, 1986) to provide a new perspective on the effects of goal-focused leadership on followers. We suggest that goal-focused leaders are able to clarify employees’ roles, thus decreasing employees’ role ambiguity and helping both themselves and their followers to perform better.

According to role theory, roles are generated by normative expectations and are related to identifiable social positions in organizational contexts (Biddle, 1986). Role theory argues that individuals’ behavior is based on “how their roles evolve and are defined” (Matta et al., 2015, p. 1692). However, when duties and role requirements are not defined clearly enough to guide the role-holder’s behavior, he or she may slump into a state termed “role ambiguity” (Biddle, 1986). Role theory suggests that role ambiguity will increase an individual’s dissatisfaction with his or her role, hesitation over decisions, anxiety and confusion, resulting in ineffective performance (Kahn et al., 1964; Rizzo et al., 1970).

Supervisors who offer goal-focused leadership are skilled in aligning individual roles with organizational goals and defining role responsibilities (Colbert and Witt, 2009). They are unlikely to abandon their responsibilities and do not exert their authority unnecessarily; they engage in active and responsive leadership behaviors and avoid the omission of reward and punishments (Hinkin and Schriesheim, 2008). Reward omission is a term used to refer to the failure to reinforce good subordinate performance and similarly punishment omission is the failure to reinforce poor subordinate performance (Hinkin and Schriesheim, 2008). Hinkin and Schriesheim (2008) proposed these concepts and demonstrated that both the two types of omission have a negative influence on role clarity. Similiarly, previous scholars argued that supervisors’ omissions of reward and punishment led to role ambiguity for followers (Rizzo et al., 1970). Given that supervisors who display a high degree of goal-focused leadership are good at clarifying and defining individual roles (Colbert and Witt, 2009), it is reasonable to infer that they may display low levels of reward and punishment omission. Hence we have drawn on role theory to suggest that leaders’ omission of reward and punishments is potentially an important potential mediator of the relationship between goal-focused leadership and employee voice. We suggest that goal-focused leadership can promote employees’ voice behavior, because leaders who display goal-focused leadership are less likely to omit reward and punishments.

**FIGURE 1:**
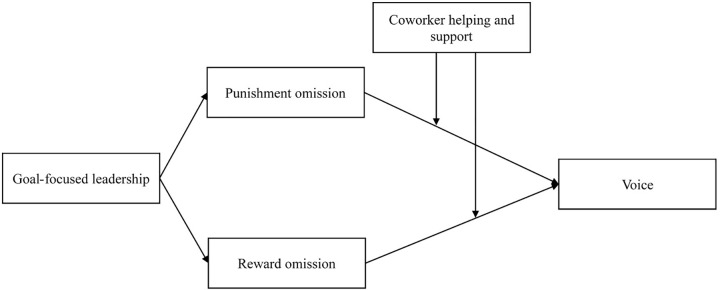
Theoretical model.

As well as recognizing the importance of leadership, role theory also proposes that coworker help and support could help employees clarify their roles and thus buffer the negative impacts of leader’s omissions. We suggest, therefore, that coworker helping and support moderate the relationship between the omission of reward and punishments and voice behavior. Coworker help and support refer to “coworkers’ assisting an employee with his or her tasks when needed by sharing knowledge and expertise or providing encouragement and support” (Zhou and George, 2001, p. 685). Previous studies suggest that coworkers are a vital part of employees’ work-life and serve an important social role as buffers against negative work outcomes such as burnout, stress and physical strains (e.g., Schwarzer and Leppin, 1989; Viswesvaran et al., 1999; Halbesleben, 2006) as well as enhancing the positive work outcomes such as performance and creativity (e.g., Zhou and George, 2001; Chiaburu and Harrison, 2008). In this study, we propose that where leaders’ fail to provide reward and punishments, coworker help and support could provide the information required to clarify their role expectations. Thus coworker help and support could buffer the negative influences of leaders’ omission of reward and punishments on employees’ voice behavior, thus maximizing the influences of goal-focused leadership on employee voice. The theoretical framework that guides the study is presented in **Figure 1**.

## Theory and Hypotheses

### Goal-Focused Leadership, Punishment Omission and Voice Behavior

Colbert and Witt (2009) argue that goal-focused leaders emphasize the importance of goal achievement, and they use policies and practices to clarify goals and ensure subordinates’ goal achievement. Goal-focused leaders are likely to understand clearly what is expected of them by the organization and what is required of them in their role as a manager. Role theory suggests that a person will know how to behave when he or she is conscious of the role expectations (Biddle, 1986). To ensure that goals are achieved smoothly, goal-focused leaders pay attention to employees’ performance and use their authority to guide followers. When employees perform poorly, a goal-focused leader will intentionally correct their behavior and direct them to the best of his or her ability. The leader may communicate goals, suggest ways of achieving them or punish poor performance; this last option is considered one of the most effective responses to employees’ poor performance (Luthans and Kreitner, 1985). Luthans and Kreitner (1985) suggested that punishment is a crucial tool for reducing undesirable employee behavior. Podsakoff et al. (2006) argued that leaders’ punishment of employees is effective because it clarifies what their leader wants them to do, thus improving their task performance. We suggest, therefore, that leaders who exhibit goal-focused leadership will be less likely to fail to respond to poor performance.

Subordinates who perform poorly will probably expect to be punished by their leader for this and so when the expected punished is not delivered they may become confused about what is expected of them (Podsakoff et al., 2006). Role theory shows that such discrepancies between expectations and reality generate negative emotions, such as anxiety and confusion, which in turn decreases engagement (Kahn et al., 1964; Bakker and Leiter, 2010; Matta et al., 2015). Consistent with role theory (Biddle, 1986; Matta et al., 2015), we argue that leaders’ punishment omission generates role ambiguity for employees and thus has a negative influence on employees’ voice behavior. Specifically, employees in this situation are likely to experience uncertainty, stress, and dissatisfaction (Rizzo et al., 1970). Spreitzer (1996) found that role ambiguity had a negative effect on individuals’ perception that they could contribute to strategic and operational performance. Employees dealing with role ambiguity are uncertain about how their behavior can contribute to the achievement of organizational goals (Fuller et al., 2006) and are therefore less likely to be committed to making constructive changes, and consequently less likely to use their voice.

Hypothesis 1a: goal-focused leadership is negatively related to leaders’ punishment omission.
Hypothesis 1b: leaders’ punishment omission is negatively related to employee voice.
Hypothesis 1c: leaders’ punishment omission mediates the relationship between goal-focused leadership and employee voice.

### Goal-Focused Leadership, Reward Omission and Voice Behavior

Goal-focused leaders are good at defining the responsibilities of individual roles (Colbert and Witt, 2009). They attach high importance to goal achievement (Colbert and Witt, 2009) and may see it as one of their important responsibilities. Using reward to promote good performance may be an effective method of fulfilling their responsibility for goal achievement, as earlier research has shown that leaders’ delivery of reward that are contingent on good performance is positively related to subordinates’ task and citizenship performance (Podsakoff et al., 2006). Role theory suggests that a person will behave on the basis of role definitions (Biddle, 1986; Matta et al., 2015). When employees demonstrate desirable behaviors that could facilitate goal attainment, a goal-focused leader will feel responsible for recognizing and reinforcing such behavior through reward. We propose that goal-focused leaders are more likely to reward good performance by employees.

The giving of reward by a leader is a form of supportive behavior and recognition of employees’ contribution to organizational goals (Jackson et al., 2012). Liang et al. (2012) argued that employees are likely to perceive speaking up as a way of reciprocating their organizations’ support and recognition. However, leaders often omit to reward followers because they do not regard it as one of their role responsibilities (Yukl, 2006; Hinkin and Schriesheim, 2008). Leaders’ reward omission has serious consequences. For example, Vroom (1964) found that leader’s reward omission resulted in a decline in employees’ motivation. Employees will be more likely to feel unfairly treated if their good performance goes unrewarded by leaders and in consequence they would not be willing to devote extra effort to even without the risks of being misunderstood and other undesirable social consequences that accompany use of voice (Morrison and Milliken, 2000). Like omission of punishment, omission of reward leads to role ambiguity (Rizzo et al., 1970). Employees will be unsure whether a behavior would be appreciated by their leaders. Role theory posits that such role ambiguity makes a person hesitate about decisions and anxious (Kahn et al., 1964; Rizzo et al., 1970) and hence more likely to remain silent rather than voicing concerns.

Hypothesis 2a: goal-focused leadership is negatively related to leaders’ reward omission.
Hypothesis 2b: leaders’ reward omission is negatively related to employee voice.
Hypothesis 2c: leaders’ reward omission mediates the relationship between goal-focused leadership and employee voice.

### Coworker Help and Support as a Moderator of the Omission –Voice Relationships

Previous research suggests that coworkers help and support colleagues by sharing knowledge and expertise, offering suggestions and guidance, and supplying task-focused feedback (Ducharme and Martin, 2000; Zhou and George, 2001). Employees who have high levels of coworker helping and support can get valuable work-related information from them when they do not get encought information from their supervisors to clarify their role expectations because he or she fails to dispense reward and punishment appropriately. Useful information from coworkers can reduce employees’ role ambiguity (Rizzo et al., 1970) so that they have a clearer understanding of what is expected of them and are less stressed and anxious. In this way the negative impact of leaders’ omission of punishments and rewards on employee voice is reduced. In contrast, lack of coworker help and support will exacerbate the role ambiguity caused by leaders’ omission of punishments and reward and thus lead to greater employee stress and anxiety and increase the negative impact of leaders’ omission of punishments and rewards on employee voice.

Hypothesis 3a: perceived help and support from coworkers positively moderates the relationship between leaders’ punishment omission and employees’ voice behavior such that the relationship is weaker when coworker help and support is high.
Hypothesis 3b: perceived help and support from coworkers positively moderates the relationship between leaders’ reward omission and employees’ voice behavior such that the relationship is weaker when coworker help and support is high.

## Materials and Methods

### Participants and Procedure

We surveyed 32 workgroups (224 employees and their 32 immediate supervisors, i.e., every workgroup included 1 supervisor and 7 subordinates) from a logistics company located in northern China. With the assistance of human-resource managers, we distributed subordinate questionnaires and supervisor questionnaires to 224 subordinates and their 32 immediate supervisor participants. No specific reward was given for questionnaire completion. Subordinate participants were asked to report their demographic information, their experience of supervisors’ goal-focused leadership and omissions of punishments and reward, as well as perceived coworker help and support; supervisor participants were asked to report their subordinates’ voice behavior. Participants completed the survey questionnaire voluntarily. Subordinate questionnaires and supervisor questionnaires were given identification codes to enable matching of responses. Respondents were assured that their responses would remain confidential. Participants sealed their completed questionnaire in the envelope provided with it and returned it 2 weeks later via a secure box outside one of the company’s meeting venues.

We excluded questionnaires with missing data, so the final sample consisted of 197 matched responses. Of the 197 subordinates, 63.5% were men (*SD* = 0.48) and the average age of participants was 29.13 years (*SD* = 5.28). 23.9% of them had a junior high school degree, 21.3% had a senior high school degree, 18.8% had a junior college degree, and 34.5% had a bachelor degree.

### Measures

#### Goal-Focused Leadership

We measured goal-focused leadership using a 5-item scale developed by Colbert and Witt (2009). A sample item is, “To what extent does [name of supervisor] provide direction and define priorities?” Response options ranged from 1, “not at all” to 7, “a lot” (α = 0.90).

#### Punishment Omission

We measured punishment omission using a 6-item scale developed by Hinkin and Schriesheim (2008). A sample item is, “I seldom get criticized by my manager when I perform poorly.” Response options ranged from 1, “never” to 7, “always” (α = 0.82).

#### Reward Omission

We measured reward omission using a 6-item scale developed by Hinkin and Schriesheim (2008). A sample item is, “I often perform well in my job and still receive no praise from my manager.” Response options ranged from 1, “never” to 7, “always” (α = 0.90).

#### Coworker Help and Support

We measured coworker help and support using a 4-item scale developed by Zhou and George (2001). A sample item is, “My coworkers willingly share their expertise with each other” Response options ranged from 1, “strongly disagree” to 7, “strongly agree” (α = 0.83).

#### Voice

We measured voice using 10-item scale developed by Liang et al. (2012). Supervisors were asked to indicated the extent to which they agreed with statements describing employees’ behavior. A sample item is, “[Employee name] proactively develops and makes suggestions for issues that may influence the unit.” Response options ranged from 1, “strongly disagree” to 7, “strongly agree” (α = 0.94).

#### Control Variables

We controlled for the participants’ age, gender, education level and duration of their experience of their leaders. Age and experience of leaders were measured in number of years. Gender was coded 1 for “male” and 2 for “female.”

## Results

### Confirmatory Factory Analysis

**Table 1** presents the confirmatory factor analysis (CFA) results. As shown, the baseline five-factor model fitted the data well (χ^2^ = 489.89; *df* = 220; RMSEA = 0.08; CFI = 0.93; TLI = 0.91). We compared the baseline five-factor model with a null model, three four-factor model, one three-factor model and one two-factor model. As shown in **Table 1**, the baseline model (five-factor model) fitted better than the null model and models 3–7, providing evidence of the construct distinctiveness of goal-focused leadership, leaders’ punishment omission, leaders’ reward omission, coworker help and support and employee voice behavior.

**Table 1:** Results of confirmatory factor analyses.

	Model	χ^2^	df	CFI	TLI	RMSEA
1	Non model	3949.18	276			
2	Baseline model	489.89	220	0.93	0.91	0.08
3	Combine reward omission and punishment omission	604.01	224	0.90	0.87	0.09
4	Combine goal-focused leadership and punishment omission	976.89	224	0.80	0.75	0.13
5	Combine goal-focused leadership and reward omission	970.30	224	0.80	0.75	0.13
6	Combine goal-focused leadership and punishment omission and reward omission	1084.39	227	0.77	0.72	0.14
7	Combine goal-focused leadership, reward omission, punishment omission and coworker helping and support	1320.86	229	0.70	0.64	0.16

### Descriptive Statistics

**Table 2** presents descriptive statistics and correlations between the studied variables. As anticipated, goal-focused leadership was negatively correlated with leaders’ punishment omission (*r* = -0.33, *p* < 0.01); and with leaders’ reward omission (*r* = -0.36, *p* < 0.01), providing preliminary support for hypotheses 1a and 2a. In addition, leaders’ punishment omission and reward omission were both negatively related to employee voice behavior (*r* = -0.19, *p* < 0.01; *r* = -0.28, *p* < 0.05), providing preliminary support for hypotheses 1b and 2b.

**Table 2:** Means, standard deviations, reliabilities, and correlations among study variables.

		Mean	*SD*	1	2	3	4	5	6	7	8	9
1	Gender	1.37	0.48									
2	Age	29.14	5.38	-0.01								
3	Education	2.69	1.22	-0.39^***^	-0.01							
4	Tenure	2.35	3.37	-0.11	0.47^***^	0.41^***^						
5	Goal-focused leadership	5.63	0.79	-0.03	-0.001	-0.09	-0.11	(0.90)				
6	Punishment omission	2.39	1.03	0.02	-0.19^***^	-0.05	0.03	-0.33^***^	(0.95)			
7	Reward omission	2.32	1.07	0.10	-0.17^**^	-0.10	0.02	-0.36^***^	0.79^***^	(0.95)		
8	Coworker helping and support	5.83	0.74	0.01	0.11	-0.08	-0.07	0.46^***^	-0.31^***^	-0.30^***^	(0.83)	
9	Voice	4.69	0.87	-0.11	0.07	0.09	-0.01	0.17^***^	-0.19^***^	-0.28^**^	0.05	(0.94)

*N = 197. Cronbach’s alpha reliabilities are in parentheses on the diagonal. ^∗^*p* < 0.05; ^∗∗^p < 0.01; ^∗∗∗^p < 0.001.*

### Hypothesis Testing

As shown in **Table 3**, goal-focused leadership was negatively associated with leaders’ punishment omission (*b* = -0.40, *p* < 0.01) and reward omission (*b* = -0.46, *p* < 0.001) after controlling participants’ gender, age, education, and working experience with leaders. Thus our hypotheses 1a and 2a were fully supported.

**Table 3:** Results of regression analysis.

	Independent variables	Punishment omission		Reward omission		Voice			
		Model 1	Model 2	Model 3	Model 4	Model 5	Model 6	Model 7	Model 8
1	Sex	-0.04	-0.08	0.13	0.08	-0.14	-0.10	-0.11	-0.07
2	Age	-0.06^***^	-0.06^***^	-0.06^***^	-0.05^***^	0.01	0.01	0.01	0.01
3	Education	-0.13^*^	-0.15^**^	-0.16^**^	-0.18^**^	0.09	0.10	0.07	0.08
4	Working experience with leaders	0.07^***^	0.06^**^	0.07^***^	0.06^**^	-0.02	-0.02	-0.02	-0.02
5	Goal-focused leadership		-0.40^***^		-0.46^***^	0.19^**^	0.20^**^	0.16^*^	0.16^*^
6	Punishment omission					-0.13^*^	-0.14^**^		
7	Reward omission							-0.20^***^	-0.23^***^
8	Coworker helping and support					-0.04	-0.06	-0.05	-0.10
9	Punishment omission × coworker helping and support						0.13^**^		
10	Reward omission × coworker helping and support								0.11^*^
	*R*^2^	0.07	0.17	0.08	0.19	0.09	0.12	0.11	0.13
	Change in *R*^2^	0.07^***^	0.10^***^	0.08^***^	0.11^***^	0.09^**^	0.03^**^	0.11^***^	0.02^*^

*N = 197; ^∗^p < 0.05; ^∗∗^p < 0.01; ^∗∗∗^p < 0.001.*

We carried out regression analysis with voice behavior as the dependent variable, entering the variables in three steps :(1) the control variables (i.e., gender, age, education level and working experience with leaders); (2) goal-focused leadership, mediators (i.e., leaders’ punishment omission and reward omission) and the moderator (i.e., coworker help and support); (3) the two-way interactive terms (i.e., leaders’ punishment omission × coworker helping and support and leaders’ reward omission × coworker help and support).

As shown in **Table 3**, leaders’ punishment omission (*b* = -0.13, *p* < 0.05; Model 5) and reward omission (*b* = -0.20, *p* < 0.001; Model 7) were significantly related to employees’ voice behavior while the relationship between goal-focused leadership was also significant (*b* = 0.19, *p* < 0.01, Model 4; *b* = 0.16, *p* < 0.05, Model 7), indicating the partial mediation role of leaders’ omission behavior. To further examine the mediation effects of leaders’ punishment and reward omission, we conducted supplementary analysis using the bootstrap method with SPSS PROCESS Macro (Hayes, 2013). With the reward omission as the mediator, the indirect effect was 0.08, 95% CI [0.029 to 0.175] and with punishment omission as the mediator, the indirect effect was 0.05, 95% CI [0.002 to 0.132]. These results provide further support for our hypotheses.

Hypothesis 3a and 3b proposed the moderating role of coworker helping and support in the relationship between leaders’ omission behavior (i.e., punishment and reward omission) and employees’ voice behavior. As shown on in **Table 3**, the coefficients of the interactive terms, leaders’ punishment omission × coworker help and support (*b* = 0.13, *p* < 0.01; Model 6) and leaders’ reward omission × coworker help and support (*b* = 0.11 *p* < 0.05; Model 8), were significant, providing support for hypotheses 3a and 3b.

**FIGURE 2:**
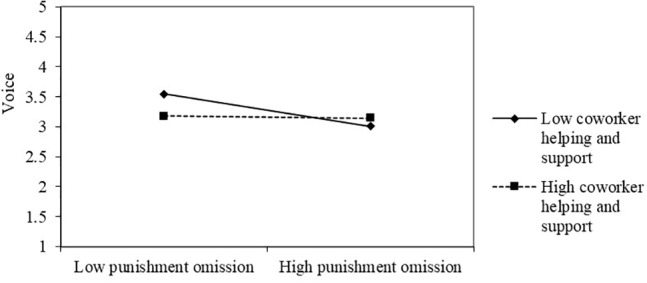
The moderating role of coworker helping and support in the relationship between punishment omission and voice.

**FIGURE 3:**
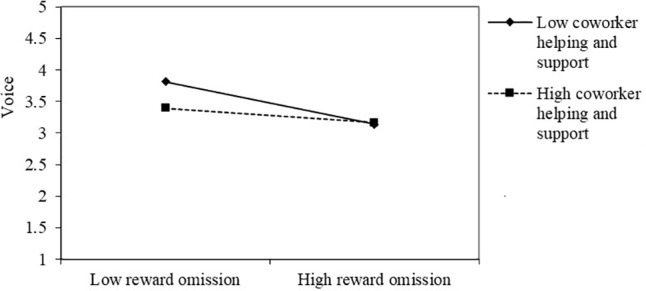
The moderating role of coworker helping and support in the relationship between reward omission and voice.

Following Cohen and Cohen (1983), we defined high coworker help and support as plus one SD from the mean and define low coworker helping and support as minus one SD. As shown in **Figures 2**, **3**. As predicted: (a) the linear relationship between leaders’ punishment omission and employees’ voice behavior was weaker for the high coworker helping and support and stronger for low coworker helping and support; (b) the linear relationship between leaders’ reward omission and employees’ voice behavior was weaker for high coworker helping and support and stronger for the low coworker helping and support.

## Discussion

In the present study, we developed and tested a model linking goal-focused leadership with employees’ voice behavior by investigating the underlying mechanisms as well as the boundary condition. We found that: (a) goal-focused leadership was positively associated with employees’ voice behavior via decreasing leaders’ punishment and reward omission; (b) perceived coworker helping and support positively moderated the relationship between leaders’ punishment omission and employees’ voice behavior in a way that the relationship was weaker when coworker helping and support is higher rather than lower; (c) perceived coworker helping and support positively moderated the relationship between leaders’ reward omission and employees’ voice behavior in a way that the relationship was weaker when coworker helping and support is higher rather than lower.

### Theoretical Implications

The present study has several theoretical implications that should be noted. First, we apply role theory (Rizzo et al., 1970; Biddle, 1986) to explain the influence of goal-focused leadership on followers. Consistent with role theory (Rizzo et al., 1970; Biddle, 1986; Matta et al., 2015), we propose that goal-focused leaders who are expert in aligning individual roles with organizational goals and defining role responsibilities will clarify their own managerial role and reduce followers’ role ambiguity, thus decreasing the stress and anxiety generated by role ambiguity and enhancing employee voice (Rizzo et al., 1970; Colbert and Witt, 2009). By applying role theory to interpret the relationship between goal-focused leadership and employee voice, the present study has enriched our theoretical understanding of how goal-focused leadership influences followers (Graen, 1976; Hobfoll, 1989; Kanfer and Ackerman, 1989; Tett and Guterman, 2000; Colbert and Witt, 2009; Perry et al., 2010; Kim et al., 2017).

Second, we have drawn on role theory (Rizzo et al., 1970; Biddle, 1986; Matta et al., 2015) to introduce a new mediator of the relationship between goal-focused leadership and employee voice, namely leaders’ reward and punishment omission. Leaders’ reward and punishment omission in the workplace has received great attention in recent years (e.g., Hinkin and Schriesheim, 2008, 2015; Arnold et al., 2015). We demonstrate that goal-focused leaders promote employee voice via decreasing their levels of omission. Most earlier research has attributed the positive effects of goal-focused leadership to its positive impact on followers’ personal development and growth, which delivers desirable outcomes (Colbert and Witt, 2009; Perry et al., 2010; Kim et al., 2017); this study offers a new perspective and extends the previous literature. We suggest that goal-focused leadership improves leaders’ performance because it minimizes non-responsive leadership behaviors and omissions of leadership behavior such as omission of reward and punishments, and in consequence it improves followers’ performance, i.e., increases their use of voice. By identifying the mediating roles of leaders’ reward and punishment omission, we have also contributed to the strand of research investigating the influencing mechanisms of goal-focused leadership (Colbert and Witt, 2009; Hülsheger and Maier, 2010; Perry et al., 2010).

Finally, by specifying the role of coworker helping and support as an important moderator on the reward and punishment omission-voice relationships, we have improved the understanding of the boundary conditions of the link between goal-focused leadership and voice behavior. Drawing on role theory (Rizzo et al., 1970), we argue that help and support from coworkers provides employees who suffer from leaders’ omission of reward and punishments vital information about role expectations, thus alleviating employees’ role ambiguity generated by leaders’ omission. Hence the effect of reward and punishment omission on voice will be weaker in the context of high coworker help and support. Our findings are consistent with studies which have identified coworkers as an important social support for employees’ extra role behaviors (e.g., Halbesleben and Wheeler, 2015; Campbell et al., 2017). We have extended this work by demonstrating that coworkers’ help and support can buffer the negative impact of ineffective managerial practice, i.e., failure to reward and punish employees appropriately.

### Practical Implications

This research has some implications for managerial practices. To begin with, our model suggests that goal-focused leadership can enhance employees’ voice behavior. This suggests that organizations should promote goal-focused leadership through training programs, to enhance leaders’ ability to encourage employees to use their voice. For instance, supervisors should link the organization’s policies to its goals, discuss goals with followers and give followers clear directions to encourage followers to voice their opinions, concerns and suggestions (Colbert and Witt, 2009).

Second, the finding concerning the mediating role of leaders’ omission of punishments and reward suggests that goal-focused leadership can promote employees’ voice behavior via minimizing leaders’ omission of punishments and reward. Leaders should therefore keep this in mind and pay extra attention to subordinates’ performance and provide appropriate feedback (Hinkin and Schriesheim, 2008). More specifically, supervisors should actively acknowledge followers’ good performance and provide positive feedback, such as praise or reward (Hinkin and Schriesheim, 2015). Similarly, managers should let subordinates know when their work does not meet expectations or the requirement and penalize them, for example by rebuking or demoting them (Hinkin and Schriesheim, 2015).

Finally, as we have demonstrated that the help and support of coworkers helps to mitigate the effects of poor leaders, strategies that encourage employees to help and support coworkers should be developed and implemented. For example, organizations could create an atmosphere of trust, as coworkers who trusts their colleagues will be more likely to provide them with help and support (Kramer, 1999; Settoon and Mossholder, 2002; Lewicki et al., 2006). Furthermore, previous research suggests that coworker helping and support behavior should be rewarded formally, meaning that organizations should have a formal system for rewarding coworkers who help and support colleagues in recognition of the benefits of such behavior (Lau and Liden, 2008; Halbesleben and Wheeler, 2015).

### Limitations

Our study has several limitations, which can be viewed as opportunities for future research efforts to improve, replicate, and extend our findings. The first limitation of the study is the cross-sectional design, which limits our ability to determine the causality of the detected associations we uncovered. Longitudinal research would be needed to do this. A second limitation is the limited generalisability of the findings. Because we collected data from just one company in China, it could be argued that our findings are contextually and culturally specific. Future research could include attempting to replicate our findings in other types of organizations and other cultures.

## Conclusion

From the perspective of role theory, our results highlight the importance of goal-focused leadership as an antecedent of voice behavior that is mediated by reward and punishment omission and the need for further research into the contingencies of this relationship. We therefore suggest that understanding of the mechanisms by which antecedents produce voice behavior would be advanced by research into the role of the mediation and moderation processes.

## Ethics Statement

All procedures performed in studies involving human participants were in accordance with the ethical standards of the institutional and/or national research committee and with the 1964 Helsinki declaration and its later amendments or comparable ethical standards with written informed consent from all subjects. This research was approved by the Human Research Ethics Committee (HREC) at Business School, Beijing Normal University.

## Author Contributions

JQ and XL substantially contributed to the conception, the design of the work as well as the preparation of the draft. BS, WZ, MC, and YQ reviewed it critically and gave important intellectual input. BW contributed to the analysis and interpretation of the data.

## Conflict of Interest Statement

The authors declare that the research was conducted in the absence of any commercial or financial relationships that could be construed as a potential conflict of interest.
